# Northern Ohio Dental Association

**Published:** 1886-06

**Authors:** F. P. Bethel


### Proceedings of the Northern Ohio Dental Association.

Reported for the DENTAL REGISTER by F. P. Bethel, D.D.S.

The 27th annual meeting of the Northern Ohio Dental Association was held in Cogswell & Co's Dental Depot, Cleveland, Ohio, Tuesday and Wednesday, May 11th and 12th, 1886.

FIRST DAY—MORNING SESSION.

The meeting was called to order at 10:45 A.M., with Dr. David Gibbons in the chair. Minutes of the previous meeting were read and approved. The treasurer's report was accepted, after which the first topic on the programme, "Diagnosis," was taken up.

*Dr. Buffett.*—To know is the foundation of all medical practice. Besides understanding the anatomy of the diseased part, we should understand that of all the parts, however remote, which may become affected.

Besides anatomy, physiology is necessary, for without both we cannot understand pathology. This is the foundation on which we build our diagnosis. The dentist is called upon to diagnose manifestations of pain connected with the teeth, namely neuralgia, and here he is expert, far above the average practitioner of medicine. Diagnosis is often difficult, as the symptoms are similar. The ordinary cases are comparatively easy, however, but we often become lost where there is slight irritation or a chronic disease of a low type.

Diseases of the bone, such as caries and necrosis, the dentist is called upon to diagnose as well as such diseases as ostitis and periostitis, pathological conditions of the antrum of Highmore, etc. Sometimes we have tumors in and about the mouth, or ulcerations of the mouth and tongue. Then there are cases of injuries to the face and fractures of the inferior maxillary presented to us, and we should be able to diagnose correctly.

*Dr. J. W. Lyder*, of Akron, cited some cases of tumors that had been placed under his care. In one case the tumor appeared of a light character, but upon thorough examination he found it to be a malignant growth, and would not operate upon it. The patient died sometime after that, and examination showed that the inflammation and absorption had been so extensive that the greater portion of one side of the maxillary bone was gone. The patient had been an opium eater for eleven years and therefore did not suffer so much from the disease. He had had cases of tumors caused by the filling up of the ducts. These were troublesome affairs, but could be successfully operated upon if one could diagnose correctly. An immediate operation on epithelioma is best. The tumor appears like a grain of corn. I have had a number of these, some small and some large. The largest, about the size of a turkey's egg, I successfully operated upon by cutting away the soft tissues and taking the bone forceps and cutting deep. In the portion removed, little threads could be seen in the bone tissue.

It has been eight years and no growth has returned. In such cases go deep and be thorough.

### AFTERNOON SESSION.

After the meeting was called to order, etc., the subject of "Diagnosis" was again taken up for discussion.

*Dr. Butler.*—This is the starting point that all professional men must recognize. In the presentation of some cases for our attention, we are called upon to exercise all the skill and tact we possess, to fathom the difficulty.

To-day the demand upon us is different from what it was a few years ago. There is a growing demand for the better preparation of men coming into the profession and no excuse for them.

Let us now take up a little item which dentists are as negligent in as any feature in practice, viz.:

"The examination of a case and the giving of advice." We should remember that it is not simply for to-day or this year, but for years to come. Take a young patient whose teeth are in malposition, and treat the case superficially so as to get it off your hands to-day, is a poor exhibition of professional skill, or advice. Two or three such cases have come under my observation within the past few days.

The great proportion that come to us need such services as are for their best interests for years to come. Take this into account and try to make as few mistakes in that direction as possible.

*Dr. F. S. Whitslar.*—No effect is the result of a single cause, but several causes may enter into the producing of a result. Symptoms are understood to be characteristic of functional or vital trouble. That which fifty years ago we called signs had reference to the physiological rather than the functional. We now have symptoms of a symptom, for instance, extreme debility as seen in dysentery, causing frequent evacuations, is a symptom of a symptom. In order to be a good diagnostician man must be born one. With the best book learning we are often in the dark.

Here he cited some cases that were difficult to diagnose.

The first was a young man's superior left molar that had been aching for some time.

The tooth was sore, but not decayed, and had no appearance of disease about it, only on the lingual side, a slight enlargement. The tartar was taken from the neck of the tooth, but did not ease it. Finally the tooth was extracted and underneath the crown, between the roots, was a well developed cyst.

The second case was that of a child, well developed, of healthy parents, who had healthy grandparents. When the first tooth appeared the mother became alarmed at an eruption on the child's face. Some of the blotches were small, and others as large as a twenty-five cent silver piece. A physician stated that it was simply tooth rash. The blotches seemingly disappeared after a tooth was erupted, and did not again appear until another tooth came through.

There was another case, similar in character, but the blotches not only appeared on the face and head, but on the wrists, and whether it was due to the eruption of the teeth, indigestion, or what, he did not profess to know, but that he would like to hear from others regarding it.

*Dr. Buffett* said it is a well-known fact, that during the eruption of the temporary teeth, there is a tendency to skin troubles. The eruption of the teeth does not make the disease, but lets down the bars and the disease takes hold. I am satisfied that three-fourths of the skin troubles are due to some hereditary disease. In this case it is impossible to tell just what the cause was unless we can get information from the parents.

*Dr. Butler* said he had never seen a case just like this, but had seen an eczemic condition of the head and wrists that was worse during the eruption of the temporary teeth.

*Dr. Brown* inquired if a scar remained after the sores healed.

*Dr. Whitslar* said none at all.

*Dr. Barnes* cited a case that came to him in February. The trouble came from the region of the left superior molar and bicuspid. A large amalgam fiilling was removed, but, he said, I found no sensitiveness in the tooth, yet it did not appear dead. I filled the cavity with gutta-percha and awaited the result. A few days after this the patient returned and stated that he had a mucous discharge from the left nostril. A physician had told him it was due to the rupture of the nerve sheath at the foramen in the antrum. I was satisfied of antrum trouble and extracted the tooth, on which, I found a large abscess, and the pulp cavity completely filled with a calcareous deposit. The patient recovered.

Another case was that of a man whose superior molar had given him some trouble. The gum was a bluish black, and the discharge of the pus through the nostrils copious.

His physician had been treating him for catarrh. Upon extraction of the tooth, I found several thimblefulls of pus in the antrum. Am still treating the case, but the patient is getting better.

*Dr. Siddall* spoke of a lady patient, whose physician had been treating her for catarrh without success.

The lady had worn full upper and under sets of teeth for years, but notwithstanding he opened up into the antrum and found a large three-rooted molar lodged in the cavity.

After extraction the patient got well.

*Dr. W. P. Horton* said it is difficult to teach others just how to diagnose. The difficulty comes from a multiplicity of causes.

We must first learn what it is to be healthy and at ease before we can understand disease. The best knowledge does not always come from book learning. Look through all the authors and you find a variety of opinions.

In regard to the case Dr. Whitslar presented no doubt it was necessary to force more blood to parts about the tooth for eruption, which caused a withdrawal from other parts, of the life-giving principles, and the disease appeared.

He said, a few years ago, when nutrition was defective, blotches appeared on his own hands, but now that his health was better, they were disappearing.

He then cited a case of a lady, whose face had become partially paralyzed on one side. The physicians said it was due to amalgam fillings in her teeth, and treated her for murcurial poisoning. The fillings proved, however, to be made of Robinson's fibrous foil, and an antopsy after death revealed a tumor at the base of the brain, that had been entirely overlooked.

*Dr. David Gibbons* said he had traced the genealogy of both sides of his family as far back as he could, and found no taint of hereditary disease, yet, when his daughter was young, an eruption came out upon her head and spread to cover the whole of that member. Its first appearance was about the time the temporary teeth began erupting. The trouble continued and was so severe he was afraid it would impair the growth of hair, but at the age of thirteen, when all but the wisdom teeth had erupted the blotches disappeared, and she has now a luxurient head of hair.

*Dr. Chas. Buffett* said there is much mistaken diagnosis. It is best not to have a hobby for we should look over the whole ground before we decide.

*Dr. W. P. Horton* cited a case of mistaken diagnosis, where the second cusp of a second bicuspid irritated the cheek, and the physician had pronounced it cancer.

The grinding down of the cusp cured the disease.

*Dr. L. P. Bethel* cited a case of mistaken diagnosis. It was that of a lady, who came to his office, with face swollen under and immediately back of the inferior maxillary. A city physician had pronounced it a well marked case of mumps, but an examination revealed a lower wisdom tooth erupting, and the tissues about it swollen. Lancing the gum over the tooth caused an effective cure.

*Dr. Fred. Lyder* said one is required to have a thorough knowledge before he can get a correct diagnosis, and as the advantages are now so great, I think Young America is the coming man.

*Dr. Butler.*—The young must necessarily come in to take the places of the older practitioners. As years go by they will find the demand for knowledge is greater and greater. There are cases presented to-day that tax our skill to the utmost. Even our best men, as Dr. Taft, Dr. Atkinson, etc., have been brought to that point where they would have given anything for a knowledge greater than they possessed. The young man can only get this knowledge by hard, earnest effort. He who strives for a patronage will get it if he perseveres. There is room enough for all; the only thing is the want of ability to meet the demand. We have all got to work with our own head and hands, and whether we do it good or ill we must be responsible as individuals. Subject passed.

EVENING SESSION.

After the usual preliminaries the subject "Different Materials for and the Methods of filling Teeth" was taken up.

*Dr. Siddall* read a paper on "Children's Teeth," a synopsis of which is presented below:

There is no knowledge that a dentist can impart that is more needed than a better general understanding of a few simple facts regarding children's teeth. Not only parents but every person of adult age—teachers, guardians, and employers of children should think and act in a sort of general way for the child.

If we knew a little more than we do and stopped to consider, we would be far more helpful to the young than we are. If we, as a society, or as individuals, could in some way get before the public mind, in the fewest words and without technical terms, enough anatomy, physiology, and hygiene, including what our profession can do to arrest the destruction of children's teeth, it would be worth our effort.

From the third until the sixth year the temporary teeth should remain in place. Then is the time of all others in life for a child to be good looking. It begins life looking about the mouth like its grandmother, or perhaps its mother, with the grinders in a bowl of water, or away to the shop for repairs; but now there is perfect harmony between the little face and little teeth. I said from the age of three to that of six the first teeth should remain intact, and the face should be symmetrical; but how is it from six to twelve or thirteen, and from that to twenty, when an entire new set of teeth shall have taken the place of the first set? It is moving time now in the poor child's mouth, so excuse him. For a while the new comers will look as if they would like to make themselves at home, if they could. A twelve year old boy will be overcome by his teeth as he would in a man's suit of clothes, only the other way.

Soon after the thirteenth year, those overgrown teeth will assume proper relations with the constantly growing face.

Would that there was no more trouble about children's teeth than that connected with their general development. It is said that everything has its enemy, its worm to destroy its symmetry.

If destruction is greater anywhere than in the child's mouth, I do not want to see it. It would seem the enemy could wait until mature age at least, if not until the natural forces begin to fail, when the eye grows dim and the ear deaf from age. But no; in the average child's mouth destruction is seen every time you look into its smiling face. You take courage, however, when the sixth year arrives, for you behold new teeth taking the place of old ones; but if you knew it all, and knew how much longer the twelve back teeth were in coming than the first set, you might not think a dentist such a scoundrel after all for going and filling baby teeth when he knew they would come out.

Four very important permanent grinders are pretty sure to put in an appearance just back of the temporary molars, a little before the front teeth begin to come out. Great wisdom is seen in this, as it secures to a child sixteen strong grinders during the few years he is changing his front teeth. But how is the average mortal to be made to understand all this, that these teeth need careful attention, that children from three to thirteen years of age should be as regular in their attendance on their dentist as the older folk, and even more so if they are to preserve their teeth in after life?

It is said that "he who knows nothing fears nothing." I think sometimes, after trying with my best efforts to have parents know something of this matter, that it might better be said: "He who fears nothing, knows nothing, and can never be made to understand."

One of our old colored bricklayers said to me once:

"Doctah, I always gits skeerd when I builds a chimbly."

When peaple 'git skeerd' over their children's teeth and 'git skeerd' in time, and keep 'skeerd' all the time, then, and only then, will the poor child have the attention from the dentist that it should have."

DISCUSSION.

*Dr. J. W. Lyder* said that he had of late given more attention to this than any other part of his practice. He had been independent and demanded the child alone if possible. If the patient was very nervous, he simply treated the tooth and dismissed the patient for that day. After operations in children's mouths, he made it a point to give the little ones some picture cards or little dishes, and notwithstanding the operation might have been quite tedious and painful, the child was always anxious to return for another operation in hopes of receiving more presents.

Where the pulp was nearly exposed, he used cement mixed with clove oil, or beech creasote, or used chloroform and Hill's stopping, applying the chloroform first. In approximal cavities facing each other, he sometimes bridged across with gutta percha or some light material where it was to be but a temporary stopping. He sometimes used amalgam, but did not believe in using gold or tin foil in the temporary teeth.

*Dr. Barnes* said, get the parents to stay at home, if possible, and let a playmate bring the child, it will stimulate her.

Gutta percha placed in the bottom of a cavity and amalgam on top makes a good filling for the temporary teeth ; yet, I am no amalgam advocate. Pulpless teeth I do not fill, but medicate as best I can.

*Dr. Colby.*—I never took kindly to amalgam. I use Weston's insoluble cement.

*Dr. Salley.*—I have kept my children's teeth in good repair with Hill's stopping, but it must be watched. A child can stand much more gutta percha on a nerve than older people.

*Dr. Gibbons* said he insisted on the use of the rubber dam, as no good filling could be made without it. The dentist should get control of the child by kindness, gentleness, and above all, firmnesss.

*Dr. F. S. Whitslar* said that cleanliness of children's teeth was very necessary. Let the mother begin to teach the child to clean its tooth as soon as one appears. If the teeth are kept scrupulously clean, it is a question if they will ever decay.

For the deciduous teeth, Hill's stopping, gutta percha, and Weston's cement are the best filling materials. Where the pulp is nearly exposed, I use cotton saturated with eugenol or cement, and iodoform $\frac{1}{4}$ parts, then creasote paste and cement. Solution of camphor and eugenol is good for the deciduous teeth. Would recommend children to use picks as well as brushes.

*Dr. Bell* said he thought that compound cavities would do better filled with cement. Coating over the filling with shellac was a good preventive of its dissolving out immediately.

SECOND DAY—MORNING SESSION.

The subject of "filling materials," etc., was again taken up and discussed.

*Dr. Gibbons.*—The only materials I find in the majority of cases to be of benefit are gold, tin, gutta percha, and occasionally, better than none, amalgam. I have never seen an amalgam filling that was a good one, although I have seen fillings stand for years. I mean that these fillings are not good for the case. I think tin foil in a soft, chalky tooth is better than gold.

Some advocate leaving a portion of decayed dentine over the pulp, but I do not have good success with this; I dress as close to the nerve as I can, and get better results.

*Dr. Dewey* used Robinson foil instead of tin, and thought it of special value at the cervical wall under gold fillings. In small cavities of children's teeth he used amalgam, but some of the plastics in large cavities.

*Dr. J. E. Bell* painted the cavity, where the nerve was nearly exposed, with iodoform, which formed a coating, then put in the plastic filling, mixing as stiff as possible, burnished and shellaced it over.

*Dr. H. F. Harvey* said that gold and tin combined in equal quantities, No. 4 gold foil and No. 3 tin, cut in strips, was a good filling material, but harder to work than gold alone.

Children's teeth he filled with tin or Robinson foil, then took a large drill, and drilling into the filling, filled it up with gold. This gave it a hard masticating surface.

*Dr. F. S. Whitslar* then read a very interesting paper on:

"How may we raise the Standard of Appreciation of Dental Services?"

The paper in substance was as follows:

The subject involved in answering the above query covers much ground. There are negative and affirmative sides to present. In considering the negative side, we introduce to you two classes, the extremes on that side of our subject. The first class of these extremes is made up of persons whe entered the profession because they thought it an easy and genteel life to live, ignorant, and too lazy to inform themselves. They have presumed enough upon the ignorance of the public in regard to dentistry to try and palm off their impudence for wisdom and ability. Their pretensions are spread broadcast by the newspapers; pretentions that shut them out from the society of the respectable portion of the profession.

The other class is made up of those individuals who fancy they have no equals; who arrogate to themselves what they do not possess, and assume positions they are not capable of filling. Men who express opinions and give advice without knowledge of facts. They seek to wrench money from the pockets of honest people. They strive to weaken the minds of people in regard to other dentists, point out defects, insinuate that the non-payment of the bill would be the proper course; suggest some alteration, or perchance a lawsuit. These pretenders are found everywhere. They count themselves sufficient for all they may utter, or any position they may occupy. These two classes of our profession, although seemingly so widely separated, are actuated by the same spirit, differing only in this: "That the narrow conception of the one promps him to promise everything for nothing; while the impudence and avarice of the other demand for nothing, everything. These, with the go-betweens constitute the millstone that hangs upon the neck of the profession, and tends to pull it down to the level of their own dishonesty, and as a result, destroy due appreciation of the value of dental services. This is one side of the subject.

In answering the subject affirmatively, we take it that the key note was struck when the American Society of Dental Surgeons held their first meeting, forty-one years ago, and declared, in the first article of their constitution, that it was their object to advance the science of dentistry by free interchange of sentiments, to give character and respectability to the profession, and also to draw the line of distinction between the truly meritorious and such as riot in the ill-gotten fruits of impudence and empiricism.

"By their fruits ye shall know them."

That there is a recognition of the value of associated effort in societies for the advancement of dentistry, I cite you to Dr. Holmes' toast to dentists, at a meeting of the New York Odontological Society:

By elevating the standard of dental education, we take the first step in raising the standard of appreciation of dental services.

Dentistry, like medicine, has had its dark days, but we think a brighter day has dawned, and that dentistry to-day, as represented by the curriculum of her colleges, stands the peer of any of the learned professions.

Although there are some now who are worthy and respected—so-called self-made men—in the profession, the time will soon come when none will be respected and patronized but the regular graduate—or if patronized, it will be only in the capacity of a quack.

A thorough and correct medical education, preceded by a good course of mental discipline and literary requirements, must henceforward be regarded as the basis of our superstructure.

Dentistry may be regarded as a specialty in medicine, but it cannot be separated from its parent stock.

Dental surgery rests on the same general principles, and is governed by the same laws that govern general surgery, hence, the same course of study is demanded in the one case as in the other. Therefore if the surgeon is a physician, a doctor, so is the dentist, the aurist, the oculist, for all are specialists in medicine.

Furthermore, to handle instruments properly the hand must be trained to do accurately whatever the mind dictates. The hand and brain must work together. The education of the eye also is of manifest importance to the artistic dentist.

A few words concerning certain virtues which conceive to be of great necessity to a dentist. Our relations to our patients are intimate, delicate, confiding in their character, and must be preserved inviolable and intact. We cannot be too particular in what occurs in the presence of patients. The dentist must be an honest, faithful, polite, intelligent, and courteous man. He should be a christian gentleman. He must be on his guard, that as few mistakes as possible occur; and the least omission of duty, or the least neglect nearly always will result in failure. Let truth gild our conversation and the character of our operations.

Let us be true to ourselves, honest to our patients, just to our fellow practitioners, and labor in love for the good of the profession.

"INCIDENTS OF OFFICE PRACTICE"

was next taken up.

*Dr. J. W. Lyder* spoke of the Robinson remedy. Said it was too strong. That equal parts of potash and carbolic acid was a superior article. When too strong, dilute with glycerine. He then spoke of poisoning from the use of rubber plates, citing some cases where a number of plates had been tried without success until a gold plate was inserted, after which the mouth got well.

*Dr. Whitslar* spoke of gold lined plates, and demonstrated the Barnes Method.

*Dr. Poole* stated that the roots of re-planted teeth would absorb. He exhibited some that had dropped out, after eight years, and the absorption had been complete.

The following officers were chosen for the ensuing year:

Dr. J. R. Bell, Cleveland, President.
Dr. John Stephan, Cleveland, Vice-President.
Dr. L. P. Bethel, Kent, O., Recording Secretary.
Dr. S. P. Dewey, Cleveland, Corresponding Secretary.
Dr. Chas. Buffett, Cleveland, Treasurer.

Adjourned to meet in Cleveland one year hence.

